# A case-based reasoning system for neonatal survival and LOS prediction in neonatal intensive care units: a development and validation study

**DOI:** 10.1038/s41598-023-35333-y

**Published:** 2023-05-24

**Authors:** Farzaneh Kermani, Mohammad Reza Zarkesh, Mostafa Vaziri, Abbas Sheikhtaheri

**Affiliations:** 1 Health Information Technology Department, School of Allied Medical Sciences, Semnan University of Medical Sciences, Semnan, Iran; 2 Maternal, Fetal and Neonatal Research Center, Tehran University of Medical Sciences, Tehran, Iran; 3 Department of Neonatology, Yas Hospital Complex, Tehran University of Medical Sciences, Tehran, Iran; 4 Independent Researcher, Semnan, Iran; 5 Department of Health Information Management, School of Health Management and Information Sciences, Iran University of Medical Sciences, Tehran, Iran

**Keywords:** Data mining, Machine learning, Neonatology

## Abstract

Early prediction of neonates' survival and Length of Stay (LOS) in Neonatal Intensive Care Units (NICU) is effective in decision-making. We developed an intelligent system to predict neonatal survival and LOS using the "Case-Based Reasoning” (CBR) method. We developed a web-based CBR system based on K-Nearest Neighborhood (KNN) on 1682 neonates and 17 variables for mortality and 13 variables for LOS and evaluated the system with 336 retrospectively collected data. We implemented the system in a NICU to externally validate the system and evaluate the system prediction acceptability and usability. Our internal validation on the balanced case base showed high accuracy (97.02%), and F-score (0.984) for survival prediction. The root Mean Square Error (RMSE) for LOS was 4.78 days. External validation on the balanced case base indicated high accuracy (98.91%), and F-score (0.993) to predict survival. RMSE for LOS was 3.27 days. Usability evaluation showed that more than half of the issues identified were related to appearance and rated as a low priority to be fixed. Acceptability assessment showed a high acceptance and confidence in responses. The usability score (80.71) indicated high system usability for neonatologists. This system is available at http://neonatalcdss.ir/. Positive results of our system in terms of performance, acceptability, and usability indicated this system can be used to improve neonatal care.

## Introduction

Neonatal mortality is considered one of the main health indicators. Mortality within the first month of life is 18 cases per every 1000 live births. Based on statistics, 2.4 million newborns (47%) in 2019 died within the first month of life^1^. A systematic review indicates that more than half of neonatal deaths occur within the first three days of life, and death within the first day is about two-thirds of this neonatal mortality rate^2^. Many of these deaths can be prevented through high-quality care and specialized post-delivery care for mothers and newborns, as well as taking care of sick or premature newborns^1^. Thus, neonatal mortality is considered very important and is known as a quality indicator for comparing the quality of care provided in Neonatal Intensive Care Units (NICUs)^3^.

Premature or sick newborns receive specialized care in NICUs, whose care period in this unit is another important challenge. The Length of Stay (LOS) of these newborns is six times as large as that of normal newborns, and in most cases, they require extensive as well as advanced care^4,5^. Their LOS elevation in NICUs increases the burden of healthcare services^4^, the level of morbidity^6^, and hospitalization costs^7^.

Accurate estimation of the risk of in-hospital mortality of newborns is necessary for the management of healthcare quality and logical use of resources^8^. Further, precise estimation of the number of days requiring intensive care and thus costs of hospitalization is important to inform parents, healthcare specialists, insurance companies, and governmental organizations for planning, and policymaking. Indeed, the physician taking care of the newborn should be able to make decisions within a short time after childbirth^5,9^. Nevertheless, decision-making on this issue is very complex, because various diagnostic and clinical factors complicate the prediction and decision-making by physicians on neonatal mortality and LOS in NICUs. In this regard, the physician should consider various factors simultaneously including the newborn status at the time of childbirth such as Birth Weight (BW), Gestational Age (GA), Apgar score, type of delivery, the maternal health condition at the time of delivery, and so on^4,10,11^. Moreover, regarding the use of resources in NICUs, there should be a combination of information about the neonates who have died or have been eventually discharged alive, whereby consideration of only one of mortality or LOS does not reflect a general view of the care provided in NICUs^12^. Nevertheless, many studies in this regard considered only one of these challenges^7,13^.

Clinical decision-making requires combining observations, and the experience of physicians with new diagnostic and specialized methods, the available clinical guidelines, and novel therapeutic strategies^14^. This can be achieved through Artificial Intelligence (AI) methods such as Case-Based Reasoning (CBR). This method has high flexibility and offers suitable performance in solving new problems based on the solutions for similar problems, and more importantly, these systems are well accepted by physicians^15^.

Many studies have been performed to predict neonatal mortality (or survival) or LOS using statistical or AI methods; for example, predicting neonates' LOS in NICUs using multivariate regression models^16^, a fuzzy expert system for predicting the risk of neonatal death^13^, K-Nearest Neighborhood (KNN), Random Forest (RF) and Bayesian network to classify the causes of fetal death^17^, decision tree (C5.0) and Artificial Neural Network (ANN) to predict neonatal mortality and preterm births in twin pregnancies^18^; However, studies on the use of CBR to predict both neonatal survival and LOS are rare. Therefore, this study was conducted to develop and evaluate a web-based CBR system that simultaneously predicts neonatal survival and LOS in NICUs.

## Related work

### Case-based reasoning

CBR is a problem-solving approach, which can employ specialized knowledge of previously-solved problems (cases). The new problem is solved by finding similar cases in the past and reusing their solutions to solve a new situation^19,20^.

The cycle of CBR is summarized in four steps:

1. Retrieval: the aim of “retrieval” is finding a case that has the greatest similarity (the most suitable) to the new problem^20^. Retrieval occurs based on applying a similarity index between the new case and all available cases in the case library. Thus, the set of cases is called retrieved cases that are ranked based on the similarity index^21,22^.
2. Reuse: at this step, the case or cases standing at the top of the list of the previous step are reused and their solutions are adapted^21^. If the situation of the new problem is exactly similar to the retrieved case, it is known as the most successful solution^20,23^. Otherwise, adoption should be used for the new problem, which can be done manually or automatically^20^. The outcome of the reuse step is creating a solution for the new case, which is called the "solved case"^21^.
3. Revise: this step involves testing the solution in a real setting or an assessment by a supervisor, a specialist, or a modeler/simulator. At the end of this step, the solved cases are considered tested or revised cases, since the system should remember only valid cases with a proper solution^24^.
4. Retaining the new case: the important feature of CBR is its learning. When a problem is solved successfully, its experience is retained in the system to solve similar problems in the future^20^; when the "revise" step creates a new case, the case base is updated with the new case (learned) to solve future problems^20,21^.

### Previous research

A Nationwide cohort study in the Netherlands used a multiple logistic regression model to estimate the risk of neonatal mortality within 28 days after birth and indicated an Area Under Curve (AUC) of 0.83^25^. Cooper et al.^26^ developed (6499 cases) and validated (3552 cases) a superleaning algorithm to predict 30-day neonatal postoperative mortality. The superlearning algorithm (14 machine learning and regression algorithms) was performed on demographic preoperative clinical data. According to the results, the superlearning algorithm outperformed all individual algorithms with regard to AUC which was 0.91 for the development and 0.87 for the validation.

In another study, researchers applied KNN, RF, and Bayesian network algorithms to classify the causes of fetal death using 49 features. The results showed that KNN and RF had the best performance (accuracy of 81.38% and 81.84%, respectively)^17^. Performance evaluation of a fuzzy expert system designed to predict neonatal mortality risk showed an accuracy (90%), sensitivity (83%), and specificity (97%)^13^.

Another study predicted the mortality and LOS for neonatal admissions to a private hospital NICU in Southern Africa using a logistic regression model. The proposed model for predicting neonatal mortality had a good fit (AUC: 0.85 and accuracy: 86.4%), but the low positive predictive value of this model reduced its performance. Furthermore, the Poisson log-linear model had a good fit (R^2^ = 0.70) for predicting LOS^9^. Coimbra et al.^27^ introduced a decision support system to predict the LOS for preterm infants using 284 cases by the CBR approach. The proposed model led to an improved "retrieval step" using optimization and the logical programming method and reduced the computational time by about 21.3% and an accuracy of 84.9%.

Rodriguez et al.^28^ proposed a prediction model for pediatric mortality risk by combining CBR, fuzzy set theory, and ANN models. After problem-solving by using the fuzzy ANN model, the CBR made justification of the solutions and stored experiences in the case base by using the KNN method (K = 3). The model was developed with 1079 cases and 33 features. Eventually, 99 cases were evaluated by seven pediatricians, which resulted in 89.89% classification accuracy.

Adawiyah et al.^29^ suggested a prediction system to detect preterm births based on CBR. For system development, 18 variables were selected and local and global similarity was determined based on Minkowsky distance. Twenty cases were used to evaluate the system in which in 18 cases, the system responses and the recorded results were similar. Hence, the system was able to detect premature infants with 90% accuracy.

## Our contribution

Generally, a variety of studies have dealt with providing a decision support system in the neonatal care domain by benefiting from artificial intelligence methods^13,17,18^. Studies on mortality, as well as LOS, have mostly focused on statistical methods and providing models^3,9,16,26,30,31^. On the other hand, we proposed a system to predict the survival and LOS of newborns in NICUs, which can be updated by adding new data (new cases) to its case base. Then, based on all data (whether at the model development or its usage) it can perform the prediction. Furthermore, the system design in this study deals with survival and LOS simultaneously. In other words, considering the importance of simultaneous prediction of survival and LOS for physicians and families of newborns, this system deals with predicting both outcomes concurrently, while previous studies have dealt with only one of these issues alone^3,13,16,26,27,31^.

Meanwhile, most previous studies have only focused on the evaluation using retrospective data without any implementation in a real setting or external validation and consideration of system acceptability and usability^3,9,13,16–18,26,27,31–33^. On the other hand, in this study in addition to the evaluation of the system with retrospective data, the system was implemented in a hospital setting and its external validation was evaluated prospectively. In addition, the usability and acceptability of proposed responses were also evaluated in a clinical setting.

## Results

### Development phase

The dataset for developing the case base contained 1346 records, which included 1225 alive (91.01%), and 121 dead (8.99%) neonates with an average LOS of 15 days (0–191 days). The distribution of the selected qualitative and quantitative features in the dataset is presented in Supplementary Table S1.

We developed the case-based system using MySQL-V5.2. The CBR process was also implemented according to the proposed architecture by weighted Euclidean distance function and KNN algorithm with PHP programming language. We also normalized the collected data using the maximum-minimum normalization method.

In the proposed CBR system, the problem-solving cycle consists of four steps; retrieval of the similar case(s) to the new problem (retrieval), using the retrieved solution to answer the new problem (reuse), reviewing the new suggested solution (revise), and maintaining the new case and using it for the future problems (retain).

- Retrieval: To find a similar case(s), the weighted Euclidean distance similarity function and the KNN algorithm were applied.
- Reuse: During the CBR cycle, the suggested solutions are presented as "Approved" or "Unapproved". In this step, the user can apply the system suggestions as a solution to the new problem ("Approved" case).
- Revise: When the proposed solution was not approved by the neonatologist, the new case is considered "Unapproved" and resolved by the neonatologist.
- Retain: Since the important part of the CBR cycle is learning from the previous cases, the solved problems are maintained in temporary tables. For this purpose, after finding and displaying the system response to the user, this response is temporarily stored in a temporary table, and after the determination of the final neonate's status in the real environment (alive/dead and LOS), the outcome is recorded in the system and transferred from the temporary table to the permanent case base.

**Figure 1:**
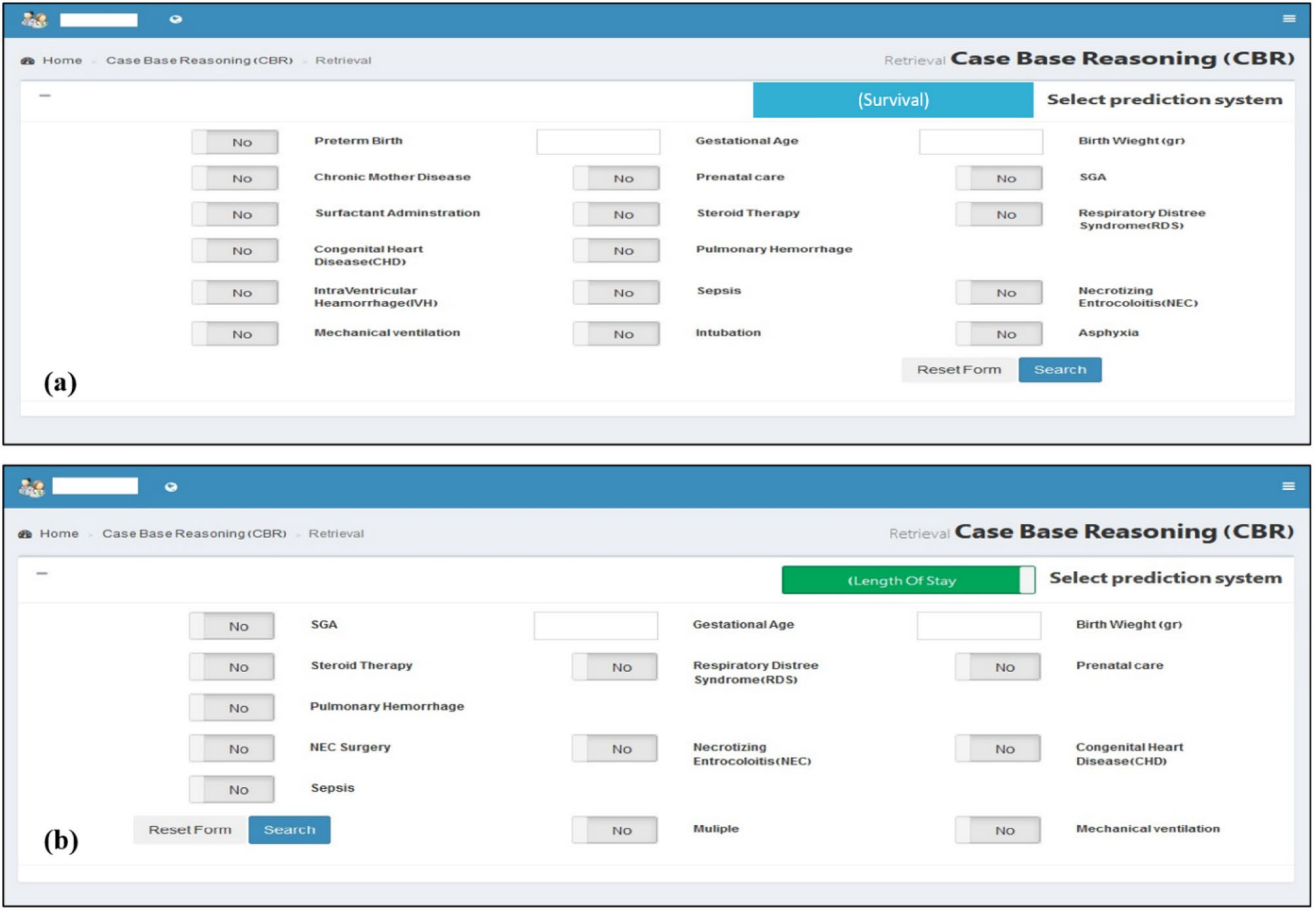
(**a**) Data entry for survival prediction system, (**b**) data entry for LOS prediction system.

**Figure 2:**
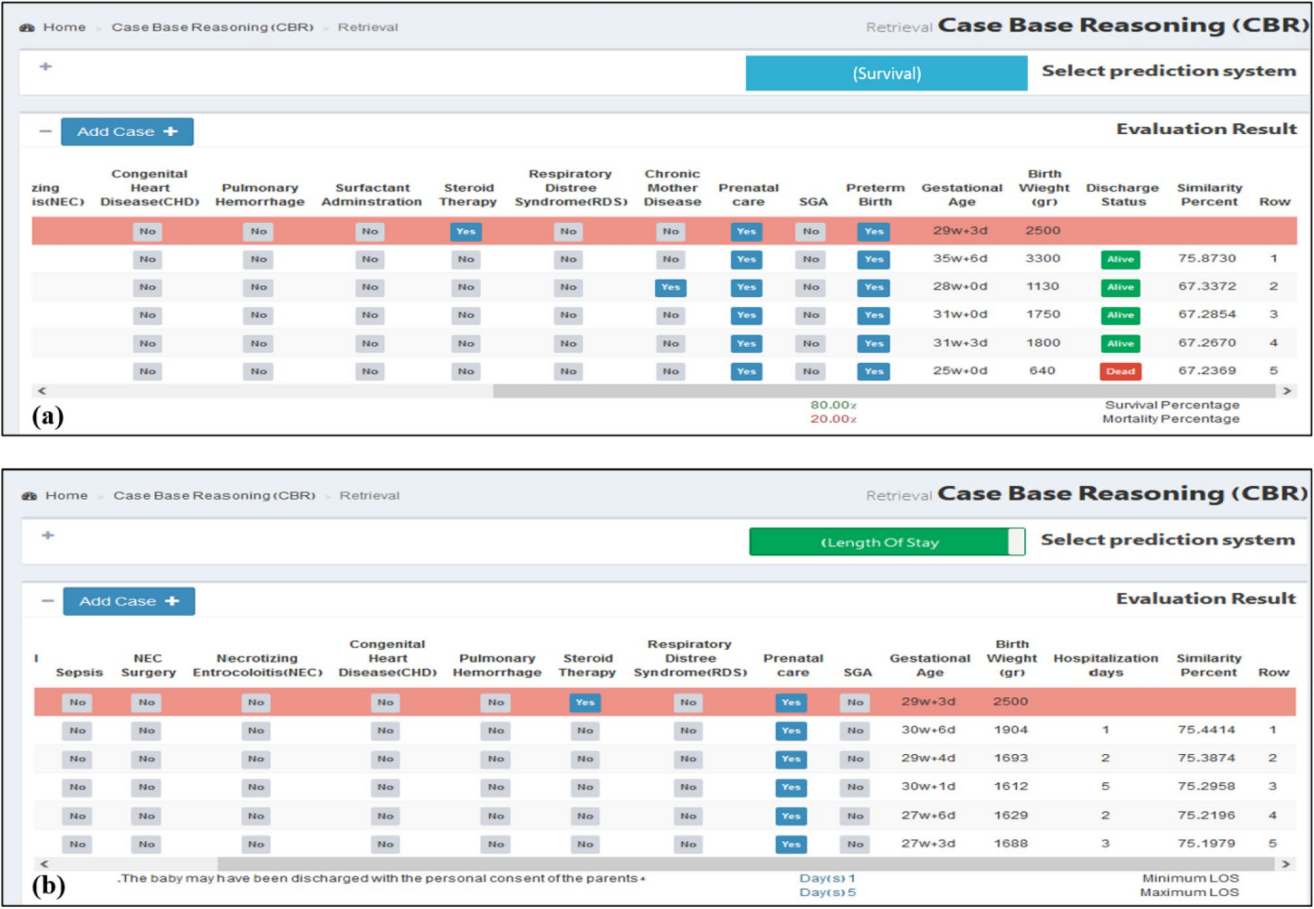
(**a**) Output view for survival prediction system, (**b**) output view for LOS prediction system.

We finally developed the web-based CBR prediction system for neonatal survival and LOS, which is available at www.neonatalcdss.ir. Figures 1 and 2 show the data entry and output views for survival and the LOS prediction system. The details related to this system are presented in sections "Evaluation phase" and "Acceptability and confidence evaluation".

### Evaluation phase

#### Retrospective evaluation

The original dataset for the evaluation included 336 records which contained 323 alive (96.13%) and 13 dead (3.87%) neonates with an average LOS of 8.5 days (0–86 days). The distribution of qualitative and quantitative variables of these neonates is presented in Supplementary Table S2.

The performance evaluation on the unbalanced case base regarding neonatal survival showed that the accuracy (97.02%), precision (98.15%), specificity (53.84%), sensitivity (98.76%), F-score (0.984), Matthews Correlation Coefficient (MCC) (0.57), and Kappa coefficient (0.624). In addition, the results of system performance on the neonatal LOS showed the RMSE was 4.79 days (Table 1). The results for the balanced case base showed an improved performance. Supplementary Tables S3–S7 shows the confusion matrix.

**Table 1:** System performance on retrospective data.

Neonatal survival system evaluation								LOS system evaluation
	Accuracy	Precision	Specificity	Sensitivity	F-score	MCC	Kappa-coefficient	RMSE
Unbalanced case base	97.02%	98.15%	53.84%	98.76%	0.984	0.57	0.624	4.79 day
Balanced case base	97.32%	98.15%	53.84%	99.07%	0.986	0.60	0.626	4.78 day

*MCC* Matthews correlation coefficient, *RMSE* root mean square error.

#### External validation

During the implementation period for the external validation, 92 neonates were admitted and included in the analysis. 74 (80.43%) neonates were finally alive, and 18 (19.57%) were dead. The average LOS was 11.39 days (1–90 days). The characteristics of these neonates regarding the selected qualitative and quantitative features are shown in Supplementary Table S8.

External validation on the unbalanced case base showed the accuracy and specificity measures were 97.82% and 88.88%, respectively. Furthermore, the kappa coefficient was 0.928 which indicated a very good agreement between the system predictions and the real outcome. In addition, the external validation for the neonatal LOS showed the RMSE was 3.49 days (Table 2). Furthermore, the system performance for the balanced case base indicated an improved performance compared to the original case base (Table 2). Given that, the external validation was performed in another hospital using prospectively collected data, the improved results show that the system can be used accurately in other healthcare centers. The confusion matrix is presented in Supplementary Tables S3–S7.

**Table 2:** System performance on prospective data.

Neonatal survival system evaluation								LOS system evaluation
	Accuracy	Precision	Specificity	Sensitivity	F-score	MCC	Kappa-coefficient	RMSE
Unbalanced case base	97.82%	97.36%	88.88%	100%	0.986	0.93	0.928	3.49 day
Balanced case base	98.91%	98.66%	94.44%	100%	0.993	0.96	0.965	3.27 day

*MCC* Matthews correlation coefficient, *RMSE* root mean square error.

### Acceptability and confidence evaluation

The acceptance and confidence levels were evaluated by using a questionnaire with a 5-Likert scale ranging from one to five (more details in “Acceptability and confidence evaluation”). The Physicians’ acceptance of survival prediction system outputs was higher than the LOS prediction system. For the survival prediction system, the mean score for acceptability and confidence were 4.88 and 4.25, respectively. Furthermore, the physicians’ acceptance and confidence in LOS prediction system responses were 4.96 and 3.96, respectively.

### Usability evaluation

Table 3 shows the completed tasks and the mean and standard deviation of the completion time for each task per second. All users performed all tasks successfully.

**Table 3:** The amount of performed tasks in each scenario.

The main task*	Subtasks	Minimum completion time (second)	Maximum completion time (second)	Mean (Standard deviation) completion time (second)
The first task (register)	4	100	277	202.6(958.2)
The second task (retrieve similar case(s) from the survival system)	5	68	201	111.6(44.0)
Third task (add new case(s) to the survival case base)	3	34	82	59.4(16.6)
Forth task (retrieve similar case(s) from LOS system)	5	20	64	44.7(14.5)
Fifth task (add new case(s) to the LOS case base)	3	18	83	41.7(20.7)

*Full details of the scenarios including the main tasks and sub-tasks are given in Supplementary Table S9.

As presented in Table 3, the longest time to perform a task was related to “registration in the system” (202.6 s). It was followed by “inputting the data related to a new case in the neonatal survival system and retrieving similar cases” (111.6 s).

Analysis of think-aloud data indicated that a set of 17 problems were identified. We categorized these problems into groups of "interface design problems", "notifications and guides", as well as "editing the elements". The interface design problems were related to adjustments to the interface and the customized profile. The problem with notifications was related to displaying the notifications pending to be verified by the system administrator for confirming the new users and the new cases. Another suggestion was adding a guideline for the data entry into the system. Further, the “editing the elements” was related to the modification of some terminologies, clarifying the measurement scales of variables, and using a single protocol at the time of data entry.

We categorized these problems and suggestions according to the Nielsen severity scale. A score of 0 means this is not a usability problem at all (2 issues), a score of 1 means cosmetic problems that do not need to be fixed unless extra time is available on the project (8 issues), a score of 2 indicates minor and low priority usability problems but are important to be fixed (4 issues), score 3 means major usability problems which are important to be fixed, so should be given high priority (3 issues), and score 4 reflects usability catastrophic problems that are imperative to be fixed before the product can be released (0 issues)^34^. In other words, more than half of the usability problems (12 issues out of 17) were due to appearance problems with low usability priority. Further, ten positive comments were provided by the users concerning the system functionalities. Furthermore, the final score of the participants for the SUS was 80.71, suggesting the high usability of the system.

## Discussion

We developed, implemented, and evaluated a CBR system to predict neonatal deaths and LOS in NICUs. Our evaluation on the retrospective data with the balanced case base indicated 0.986 and 4.78 days for F-score and RSME, respectively. The results of external validation on the balanced case base indicated 0.993 for the F-score and 3.27 days for the RMSE. In another study that was conducted on the same dataset, different feature selection methods like neonatologists' opinions, and statistical and machine learning methods were performed, and the results showed that the neonatologists' opinions resulted in a better performance^35^. Therefore, we implemented the CBR system based on features selected by neonatologists.

In another study, the accuracy of a CBR system to detect preterm labor and premature births was 90%^29^. Jaskari’s study^36^ for predicting neonatal mortality had a good AUC (0.922) and an F-score (0.477) for the RF classifier. Cooper et al.^26^ introduced a postoperative mortality risk prediction; their model AUC for the development and validation phases was 0.91 and 0.87, respectively. Beluzos et al.^37^ developed a new decision-support method for classifying neonates based on neonatal mortality risk and obtained accuracy and AUC of 93% and 0.965, respectively. Other researchers developed a fuzzy expert system to predict neonatal mortality risk with 90% accuracy^13^. A decision tree-based decision support system (DSS) was reported with 63.24% sensitivity and 99.95% specificity for predicting mortality after 10 min, and 63.24% sensitivity and 91.97% specificity for twin pregnancy deaths^18^. A pediatric death risk prediction was introduced by combining CBR, ANN, and fuzzy methods with 89% accuracy^28^. The accuracy, F-score, and sensitivity measures for predicting mortality in these studies were lower than in our study. In Sheikhtaheri’s study^35^, which was conducted on the same data as the current study, ANN outperformed other machine learning models; however, their ANN has a lower performance compared to our suggested CBR system.

The accuracy of the LOS prediction system provided by Coimbra et al. was 84.9%^27^. Pepler’s suggested model had an AUC and accuracy of 0.85 and 86.4%, respectively for death prediction and R^2^ = 0.70 for LOS prediction^9^. Among the reviewed studies, only in one study^28^, the system has been implemented in a real environment and performed external validation. Table 4 summarizes the related studies.

**Table 4:** Comparison of our results with the related literature.

Author/ year (reference)	Domain	Applied model	Evaluation sample/ number of features attribute	Evaluation Method	Performance evaluation
Adawiyah, 2020^29^	Preterm birth	CBR	20 test data/18	Retrospective/internal validation	Accuracy: 90%
Jaskari, 2020^36^	Neonatal Mortality Morbidity	LR, LDA, QDA, KNN, SVM, 3 different Gaussian process, RF	977 cases/10	Retrospective/internal validation	AUC (RF): 0.922 F-Score (RF): 0.47
Cooper, 2018^26^	Neonatal mortality	14 machine learning algorithms & LR	3552 cases/68	Retrospective/internal validation	Best results in 14 machine learning algorithms AUC (development): 0.91 AUC (test): 0.87
Beluzos, 2020^37^	Neonatal mortality	XGBoost, LR, RF	698 cases/23	Retrospective/internal validation	Accuracy (RF): 93% AUC (RF): 0.965
Coimbra, 2016^27^	LOS	CBR	284 cases/13	Retrospective/ internal validation	Accuracy: 84.9%
Safdari, 2016^13^	Neonatal mortality	Fuzzy logic	200 records/14	Retrospective/internal validation	Accuracy: 90% Specificity: 83% Sensitivity: 97%
Gunaratnam, 2013^18^	Mortality- preterm birth	Decision tree (C5.0) & ANN	32,760 cases/13	Retrospective/internal validation	Best results for Decision tree (mortality) sensitivity: 62.24%, precision: 99.95% (for preterm birth) sensitivity: 79.32%, precision: 91.97%
Pepler, 2012^9^	Neonatal mortality-LOS	LR	1578 cases/10	Retrospective/internal validation	AUC: 0.85 Accuracy: 86.4% R2: 0.70
Rodríguez, 2008^28^	Pediatric mortality	Combined CBR, ANN, and Fuzzy logic	99 cases/33	Prospective/external validation	Accuracy: 89.89%
Sheikhtaheri, 2021^35^	Neonatal mortality	ANN, decision tree, SVM, Bayesian Network, and Ensemble models	92 cases/17	Prospective/external validation	Accuracy: 86% Precision: 96% Specificity: 83% Sensitivity: 86% F-score: 0.91 AUC: 0.92
Our results	Neonatal survival -LOS	CBR	Retrospective evaluation on the 336 cases, prospective evaluation on 92 cases/17 for survival, 13 for LOS	Retrospective and Prospective/internal and external validation	Retrospective on the balanced dataset: F-score for survival: 0.986, RSME for LOS: 4.78 Prospective on the balanced dataset: F-score for survival: 0.933, RSME for LOS: 3.27

*CBR* case-based reasoning, *LR* logistic regression, *LDA* linear discriminant analysis, *QDA* quadratic discriminant analysis, *KNN* K-nearest neighborhood, *SVM* support vector machine, *RF* random forest, *ANN* artificial neural network, *AUC* area under curve, *RMSE* root mean square error.

One of the factors affecting the approval of DSSs is the users’ acceptance and a usable user interface. In this regard, cognitive methods have gained popularity for identifying system usability problems. Meanwhile, an important prerequisite in designing an effective user interface is minimizing cognitive demands^38^. In this study, according to the Nielsen scale, more than half of the usability problems (12 out of 17 cases) were due to the appearance of the system with low priority. Further, the SUS score (80.71) indicates that the system is considered usable and user-friendly by neonatologists.

In addition, the evaluation of the system acceptability suggested that neonatologists were satisfied and confident in the outputs of the system for most cases. Other researchers evaluated the usability of a DSS for antibiotic prescription in NICUs^39^, mobile applications for perinatal period health^40^, and mobile applications for pregnant women^41^ and highlighted the importance of system usability in this field. For example, in line with our results, physicians indicated the importance of the appearance and design of the user interface of a DSS for prescribing antibiotics^42^. Other usability evaluations of CBR systems indicated the importance of learnability, memorability^43^, ease of use, and confidence in these systems^44^. These studies suggest that CBR systems are accepted by physicians if they are designed properly.

The main audience of this study is neonatologists, who through the system can make decisions on determining the outcomes of neonates at the time of admission in NICUs. In addition, the use of the system to identify neonates at risk of death allows for developing a specialized team for better decision-making and providing advanced care for neonates at higher risk. Moreover, healthcare providers and hospital managers would be able to allocate and plan properly for hospital resources to manage NICU beds and workload. Application of this system can lead to an improved notification to parents about the duration required for their babies’ hospitalization in NICU as well as the healthcare costs plus notification to them about the neonate’s outcome as well as their psychological preparation.

Some limitations should be considered. The number of dead neonates was far lower than the live ones. Despite creating artificial data for the mortality class, “specificity” was low for the retrospective data. However, external validation suggested an improved performance of the system for predicting neonate survival and LOS. Further, the small number of samples for the external validation was due to the implementation of this system in only one hospital in 3 months which is another limitation of this study. It is suggested that the system should be implemented in more hospitals and evaluated with more samples. In addition, we applied simple methods (mean, median, and mode replacement) to impute the missing values, it is suggested that other researchers apply more advanced imputing methods in future studies.

In conclusion, we introduced a web-based CBR system for predicting neonatal death and LOS in NICUs. The evaluation showed that the system has a good performance in predicting neonatal survival and LOS based on similar cases. Moreover, the system outputs are mostly acceptable and trustable.

## Methods

### Study design and settings

This study was performed in Tehran, Iran on NICU-admitted neonates. To design the system, we used the data available in the "Maternal, Fetal, and Neonatal Research Center" as an academic center. External validation of the system was performed prospectively with the admitted neonates to the NICU of “Yas” hospital affiliated with Tehran University of Medical Sciences (TUMS).

### Development phase

#### Feature selection and weight calculation

To identify risk factors, we conducted a systematic review published elsewhere^11^. We also considered another systematic review on LOS predictors in NICUs published in 2015^4^, and other studies published after this review^3,12,16,31,45–50^ to identify LOS predictors.

We asked the neonatologists about the importance of these risk factors using a 5-point scale questionnaire (lowest to highest importance). The questionnaire consisted of 47 identified risk factors for neonatal death and 37 ones for LOS. It was distributed among all 30 neonatologists in Tehran, Iran and finally was completed by 22 individuals (73% response rate). A minimum of 60 percent agreement on the importance (very important and important) of each variable was considered as the selection criteria. Based on our analysis, 20 and 14 risk factors were considered important for the prediction of neonatal mortality and LOS, respectively, but some of them were not recorded in the dataset (1 variable: mother infection) and were excluded. In addition, 3 variables (septic shock, septicemia, and prematurity) had high missing values (more than 70%); therefore, we did not extract data about these variables.

Accordingly, risk factors for neonatal mortality include 17 items including steroid use, surfactant administration, mother chronic disease, prenatal care, preterm birth, BW, small for gestational age (SGA), respiratory distress syndrome (RDS), asphyxia, GA, sepsis, congenital heart disease (CHD), mechanical ventilation, intubation, intraventricular hemorrhage (IVH), necrotizing enterocolitis (NEC) and pulmonary hemorrhage. Furthermore, 13 selected risk factors for LOS prediction in NICU included steroid use, prenatal care, BW, GA, SGA, RDS, sepsis, CHD, mechanical ventilation, NEC, NEC therapy, pulmonary hemorrhage, and multiple gestations.

Moreover, we considered the mean value of each variable as the weights assigned by the neonatologists to design the CBR system. We normalized these weights using the linear normalization method between 0 and 1^51,52^. Table 5 lists the neonatal mortality and LOS predictors along with the normalized weights.

**Table 5:** Neonatal mortality and LOS risk factors with normalized weights.

Mortality risk factors (normalized weight)
Asphyxia (0.0631), BW (0.0645), CHD (0.0602), GA (0.0631), Intubation (0.0545), IVH (0.0545), Mechanical ventilation (0.0573), Mother chronic disease (0.0545), NEC (0.0588), Prenatal care (0.0559), Preterm birth (0.0659), Pulmonary hemorrhage (0.0588), RDS (0.0588), Sepsis (0.0602), SGA (0.0573), Steroid use (0.0545), Surfactant administration (0.0573)
LOS risk factors (normalized weight)
BW (0.0837), CHD (0.0819), GA (0.0837), Mechanical ventilation (0.0744), Multiple gestation (0.0726), NEC (0.0800), NEC therapy (0.0763), Prenatal care (0.0707), Pulmonary hemorrhage (0.0856), RDS (0.0744), Sepsis (0.0782), SGA (0.0707), Steroid use (0.0670)

*BW* birth weight, *CHD* congenital heart disease, *GA* gestational age, *IVH* intraventricular hemorrhage, *NEC* necrotizing enterocolitis, *RDS* respiratory distress syndrome, *SGA* small for gestational age.

#### Dataset description and data preprocessing

To develop the system, we used data related to NICUs discharged neonates (alive/dead) from a neonatal registry database from "The Maternal, Fetal, and Neonatal Research Center", TUMS. The inclusion criteria were admitted neonates to the NICUs who were born in the same hospital; therefore, transferred cases to the hospital due to surgery and other reasons, and readmission cases were excluded. The extracted data was from 6 July 2017 to 6 July 2018 and included 1682 records. We randomly selected 80% of the data to develop the case base (1346 records, 1225 alive, and 121 dead).

We developed the survival and LOS case bases separately based on the identified features. The selected features consisted of quantitative and qualitative data. Quantitative variables included BW and GA, and other variables were in the form of "Yes" or "No" values. In general, the missing data were seen in 16 of the variables; the highest rate was for NEC therapy (1.07%) and the lowest rate was for RDS (0.05%). Imputing the missing value was done according to the distribution of that variable in that class. It means that the mean for the death class and the alive class was applied separately, not the mean of the entire dataset. Also, if the data distribution was normal, we used the mean, otherwise, the median was used. As for Boolean data, we considered the distribution of values (ratio) in that variable in each class (not the ratio in the entire dataset). Details on the frequency of missing data in different features are presented in Table 6.

**Table 6:** Frequency of missing data in different features.

Variables	Missing data in dead class (N)	Missing data in alive class (N)	Total
Birth weight (BW)	1	8	9
Gestational age (GA)	2	10	12
Prenatal care	0	2	2
Mother disease	1	1	2
Steroid therapy	1	1	2
Surfactant administration	1	1	2
Pulmonary hemorrhage	1	1	2
Congenital malformation	0	2	2
Multiple gestations	2	0	2
Necrotizing Enterocolitis (NEC)	1	1	2
NEC therapy	5	13	18
Sepsis	0	2	2
Intra ventricular hemorrhage (IVH)	1	1	2
Asphyxia	1	1	2
Intubation	1	1	2
Respiratory distress syndrome (RDS)	0	1	1

Furthermore, the frequency of records in each class was imbalanced, so that the "dead" to "alive” class ratio was 1 to 10 (only 121 records for the dead class). Therefore, we balanced the data using the synthetic minority over-sampling technique (SMOTE) method to develop the system^53^.

### Proposed CBR architecture

**Figure 3:**
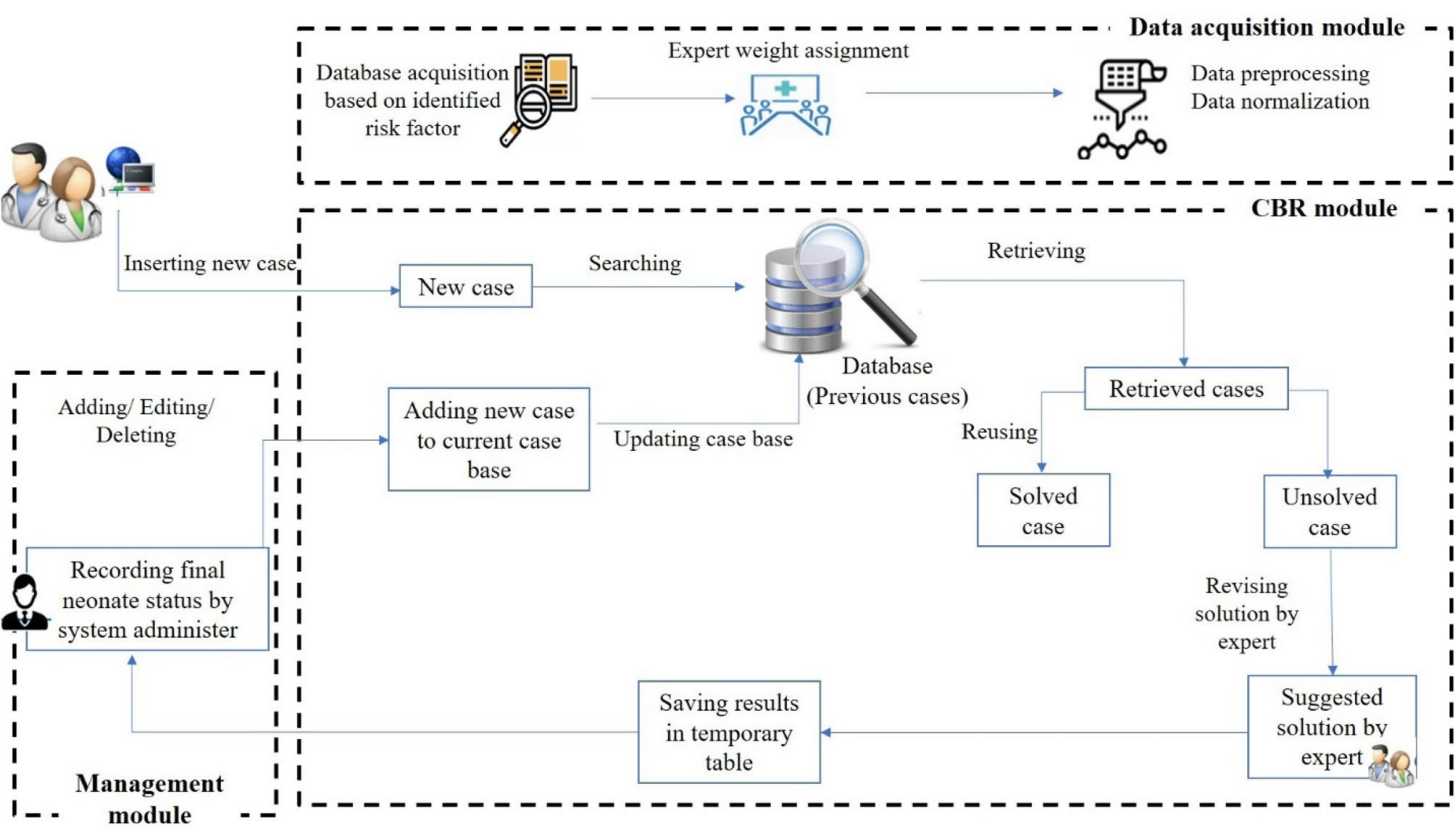
CBR system architecture for predicting neonatal survival and LOS in the NICU.

After reviewing the existing articles and architectures for CBR systems, we developed an initial architecture^54–56^. The proposed architecture was discussed in an expert panel meeting with neonatologists, and accordingly, the final architecture for CBR predicting system for neonatal survival and LOS at NICUs was developed (Fig. 3).

As shown in Fig. 3, the final architecture consists of three modules: data acquisition module, CBR module, and management module. In the data acquisition module, similar to the proposed architecture by Lin et al.^57^, developing the database is performed based on the determined variables for mortality and LOS and experts assign the weights. After the data normalization process, we developed the case base of the CBR system which includes the previously solved cases. The system administrator can update the case base through the data acquisition module.

After a user enters a new case, the CBR module retrieves similar cases based on the previous cases in the case base (search process in the case base) using the weighted Euclidean distance similarity function (Eq. 1) and KNN algorithm (K is determined by the user).

1 $$\mathrm{Weighted \;Euclidean \;Distance}= \sqrt{\frac{\sum {\mathrm{w}}_{\mathrm{i}}^{2}{\left({\mathrm{x}}_{\mathrm{i}}-{\mathrm{y}}_{\mathrm{i}}\right)}^{2}}{\sum {\mathrm{w}}_{\mathrm{i}}^{2} }}$$

$$\sum {w}_{i}^{2}: The \;sum \;of \;squared \;weights$$

$${x}_{i}: New \;case$$

$${y}_{i}: Previous \;case$$

The percentage of similarity between each of the retrieved cases with the new case is also shown to the user. In these conditions, similar to various studies^58–60^, two solution states "solved" and "unsolved" are considered for the new problem. If based on the similarity percentage and the specialist opinion, the retrieved cases match the new problem, one of the system solutions (previously solved cases) is determined as the solution to the new problem, and then that solution is regarded as verified. On the other hand, if the retrieved solutions cannot be used for the new problem, the new case solution is considered unapproved. Then, similar to some studies^55,61^, the specialist predicts the status of the hospitalized newborn (alive/dead and LOS) according to his/her knowledge and experience, and after his/her verification, the solution to the new problem is stored in a temporary table. Eventually, once the conditions of the neonate become clear (real discharge status: alive/dead and LOS), similar to previous studies^23,54,55,57,58,61,62^, the system administrator registers the final status of the new cases based on the neonate medical record in the system. With his/her final verification, these cases would be transferred from the temporary case base to the main case base and would be stored there. In the management module, cases can be added, deleted, or edited by the administrator.

### Evaluation phase

#### Retrospective evaluation

Before the web-based system implementation, the system performance was evaluated using retrospective data. For this purpose, we used 20% of the total collected data (336 records) and compared the results of the system with the actual patients’ outcomes.

#### Prospective evaluation and external validation

To perform an external evaluation, we implemented the system for 3 months (20 April 2020 to 20 July 2020) in the NICU of "Yas" hospital affiliated with TUMS. During this period, 92 neonates were admitted and their data from admission time was entered into the system by their physicians and the system predictions were recorded into the system. We followed up the neonates until discharge, and after determining the neonatal status at discharge, we recorded the actual outcomes and compared them with the system prediction.

### Acceptability and confidence evaluation

During the system implementation, we evaluated the acceptability of the system responses by the physicians in the NICU of "Yas" hospital (including three neonatologists and four pediatricians). We evaluated the acceptance level using a researcher-made questionnaire that consisted of two questions to assess physicians' acceptance and confidence level with the system's responses. Questions were on a 5-Likert scale ranging from one (very low) to five (very high). After using the system for each neonate, physicians recorded the system predictions and also their acceptance and confidence level in the system predictions.

### Usability evaluation

We evaluated the system usability both quantitatively and qualitatively. For the qualitative evaluation, we applied the think-aloud method. The users were asked to perform the tasks developed by the researchers and a neonatologist based on specific scenarios and then expressed their opinions through the think-aloud method. Completion of the scenarios and users’ opinions was recorded audio-visually using a recorder, video capturing camera, and Camtasia software.

During the usage of the system, the researcher also asked questions from the users to encourage them to continue thinking aloud. The scenarios included five tasks (Supplementary Table S9). Each task involved a set of subtasks ranging between three to five activities. If all subtasks related to the main task were performed completely, it would be considered a successful task, while performing some parts of a task was not acceptable.

We also used the System Usability Scale (SUS) questionnaire to measure the general system usability. SUS includes 10 items as five options, with the response to them, ranked from 1 (absolutely disagree) to 5 (absolutely agree)^63^. The reliability and validity of the SUS have already been confirmed^64^.

### Data analysis

We used Statistical Package for the Social Sciences (SPSS) software version 20 to analyze the data. We analyzed the data using a confusion matrix and performance measures including accuracy, precision, sensitivity, specificity, F-score, MCC, and Kappa coefficient based on nearest neighbor (K = 1)^65^ to determine the performance of the neonatal survival system, and Root Mean Square Error (RMSE) measure for LOS prediction system. We applied standard formulas to calculate these metrics (Supplementary Tables S3–S7).

The acceptability of the system was calculated using the mean of scores. We scored responses from one (very low) to five (very high) and calculated the mean of scores obtained from users.

We also applied descriptive statistics to analyze quantitative data from the SUS questionnaire based on standard methods. The final score is between zero and 100. A score of 70 or higher indicates that the system is usable, with 50 to 70 as the boundary point and less than 50 indicates that the system is unacceptable in terms of usability^64,66^.

We transcribed and analyzed the participants’ opinions from think-aloud using the content analysis method and organized the opinions. Furthermore, we used the Nielsen severity scale^34^ to classify the usability problems. To this end, all of the identified problems were discussed and classified by the research team. Finally, we calculated the time duration of the performed actions.

### Ethics declarations

This study received ethical approval from the research ethics committee of Iran University of Medical Sciences, Tehran, Iran (IR. IUMS.REC1396.9321481001). The study was performed in compliance with the World Medical Association Declaration of Helsinki on Ethical Principles for Medical Research Involving Human Subjects, and the research regulations of the country.

Considering the retrospective nature of the study, Informed consent was waived by the ethics committee of the Iran University of Medical Sciences.

## Supplementary Information

Supplementary Information.

## Data Availability

The data are not available because of the confidentiality policy of the neonatal registry of the Maternal, Fetal, and Neonatal Research Center. Data are however available from the authors upon reasonable request and with permission of the Maternal, Fetal, and Neonatal Research Center. The corresponding author should be contacted in case one needs to access the data.
