# Supplementary material for: A case-based reasoning system for neonatal survival and LOS prediction in neonatal intensive care units: a development and validation study
Source: Sci Rep. 2023 May 24;13:8421. doi: 10.1038/s41598-023-35333-y (PMC10209210; doi:10.1038/s41598-023-35333-y)
Supplement: Supplementary file 1 — Supplementary Information. [file 41598_2023_35333_MOESM1_ESM.docx]

# **Supplementary Tables**

**Table S1**. Distribution of qualitative and quantitative features in the unbalanced dataset

| **Variables** | **Values** | **Survived**  **(Mean ± SD)** | **Dead**  **(Mean ± SD)** | **All neonates**  **(Mean ± SD)** | **Missing value**  **N (%)** |
| --- | --- | --- | --- | --- | --- |
| **Quantitative features** | | | | |  |
| Birth Weight (BW) | 400-6509 | 2530.3**±**906 | 1638.8±909.4 | 2450.1±909.4 | 6 (0.4) |
| Gestational Age (GA) | 155-295 | 248±26.8 | 215.8 ±27. 0 | 245.3±27.0 | 10 (0.7) |
| **Qualitative features** | | | | |  |
| **Variables** | **Values** | **Survived**  **N (%)** | **Dead**  **N (%)** | **All neonates**  **N (%)** | **Missing value**  **N (%)** |
| Preterm birth | Yes  No | 689 (56.2)  536 (43.8) | 100 (82.6)  21 (17.4) | 789 (58.6)  557 (41.4) | 0 (0) |
| Small for Gestational Age (SGA) | Yes  No | 246 (20.1)  979 (79.9) | 39 (32.2)  82 (67.8) | 285 (21.2)  1061 (78.8) | 0 (0) |
| Prenatal care | Yes  No | 1186 (96.8)  39 (3.2) | 117 (96.7)  4 (3.3) | 1303 (96.8)  43 (3.2) | 2 (0.1) |
| Mother disease | Yes  No | 464 (37.9)  761 (62.1) | 41 (33.9)  80 (66.1) | 505 (37.5)  1395 (62.5) | 1 (0.07) |
| Multiple birth | Yes  No | 186 (15.2)  1039 (84.8) | 27 (22.3)  94 (77.7) | 213 (15.8)  1133 (84.2) | 1 (0.07) |
| Respiratory Distress Syndrome (RDS) | Yes  No | 389 (31.8)  836 (68.2) | 65 (53.7)  56 (46.3) | 454 (33.7)  892 (66.3) | 1 (0.07) |
| Steroid therapy | Yes  No | 5 (0.4)  1220 (99.6) | 1 (0.8)  120 (99.2) | 6 (0.4)  1340 (99.6) | 0 (0) |
| Surfactant administration | Yes  No | 280 (22.9)  945 (77.1) | 77 (63.6)  44 (36.4) | 357 (26.5)  989 (73.5) | 2 (0.1) |
| Pulmonary hemorrhage | Yes  No | 1221 (99.7)  4 (0.3) | 24 (19.8)  97 (80.2) | 1245 (92.5)  101 (7.5) | 2 (0.1) |
| Congenital malformation | Yes  No | 276 (22.5)  949 (77.5) | 57 (47.1)  64 (52.9) | 333 (24.7)  1013 (75.3) | 2 (0.1) |
| Necrotizing EnteroColitis (NEC) | Yes  No | 26 (2.1)  1199 (97.9) | 6 (5.0)  115 (95) | 32 (2.3)  1314 (97.7) | 0 (0) |
| NEC therapy | Yes  No | 2 (0.2)  1223 (99.8) | 0 (0)  121 (100) | 2 (0.1)  1344 (99.9) | 15 (1.1) |
| Sepsis | Yes  No | 569 (46.5)  656 (53.5) | 50 (41.3)  71 (58.7) | 619 (46)  727 (54) | 2 (0.1) |
| Intra Ventricular Hemorrhage (IVH) | Yes  No | 202 (16.5)  1023 (83.5) | 82 (67.8)  39 (32.2) | 284 (21.1)  1062 (78.9) | 2 (0.1) |
| Asphyxia | Yes  No | 22 (1.8)  1203 (98.2) | 5 (4.1)  116 (95.9) | 27 (2)  1319 (98) | 1 (0.07) |
| Intubation | Yes  No | 80 (6.5)  1145 (93.4) | 70 (57.9)  51 (42.1) | 150 (11.1)  1196 (88.9) | 2 (0.1) |
| Ventilation | Yes  No | 143 (11.7)  1082 (88.3) | 91 (75.2)  30 (24.8) | 234 (17.4)  1112 (82.6) | 0 (0) |

**Table S2**. Distribution of qualitative and quantitative features in the retrospective dataset for CBR evaluation

| **Variables** | **Values** | **Survived**  **(Mean ± SD)** | **Dead**  **(Mean ± SD)** | **All neonates**  **(Mean ± SD)** | **Missing value**  **N (%)** |
| --- | --- | --- | --- | --- | --- |
| **Quantitative features** | | | | |  |
| Birth Weight (BW) | 660-4850 | 2751.8±772.2 | 1897±781.2 | 2718±772 | 2 (0.6) |
| Gestational Age (GA) | 175-295 | 256±20.7 | 227±20.4 | 255±21 | 1 (0.3) |
| **Qualitative features** | | | | |  |
| **Variables** | **Values** | **Survived**  **N (%)** | **Dead**  **N (%)** | **All neonates**  **N (%)** | **Missing value**  **N (%)** |
| Preterm birth | Yes  No | 146 (45.2)  177 (54.8) | 8 (61.5)  5 (38.5) | 154 (45.8)  182 (54.2) | 0 (0) |
| Small for Gestational Age (SGA) | Yes  No | 56 (17.3)  267 (82.7) | 4 (30.8)  9 (69.2) | 60 (17.9)  276 (82.1) | 0 (0) |
| Prenatal care | Yes  No | 320 (99.1)  3 (0.9) | 11 (84.6)  2 (15.4) | 331 (98.5)  5 (1.5) | 0 (0) |
| Mother disease | Yes  No | 130 (40.2)  193 (59.8) | 7 (53.8)  6 (46.2) | 137 (40.8)  199 (59.2) | 1 (0.3) |
| Multiple birth | Yes  No | 41 (12.7)  282 (87.3) | 4 (30.8)  9 (69.2) | 45 (13.4)  291 (86.6) | 1 (0.3) |
| Respiratory Distress Syndrome (RDS) | Yes  No | 73 (22.6)  250 (77.4) | 9 (69.2)  4 (30.8) | 82 (24.4)  254 (75.6) | 0 (0) |
| Steroid therapy | Yes  No | 1 (0.3)  322 (99.7) | 0 (0)  13 (100) | 1 (0.3)  335 (99.7) | 2 (0.6) |
| Surfactant administration | Yes  No | 41 (12.7)  282 (87.3) | 10 (76.9)  3 (23.1) | 51 (15.2)  285 (84.8) | 0 (0) |
| Pulmonary hemorrhage | Yes  No | 0 (0)  323 (100) | 6 (46.2)  7 (53.8) | 6 (1.8)  330 (98.2) | 0 (0) |
| Congenital malformation | Yes  No | 43 (13.1)  280 (86.7) | 6 (46.2)  7 (53.8) | 49 (14.6)  287 (85.4) | 0 (0) |
| Necrotizing EnteroColitis (NEC) | Yes  No | 4 (1.2)  319 (98.8) | 0 (0)  13 (100) | 4 (1.2)  332 (98.8) | 2 (0.6) |
| NEC therapy | Yes  No | 0 (0)  323 (100) | 0 (0)  13 (100) | 0 (0)  336 (100) | 3 (0.9) |
| Sepsis | Yes  No | 154 (47.7)  169 (52.3) | 4 (30.8)  9 (69.2) | 158 (47)  178 (53) | 0 (0) |
| Intra Ventricular Hemorrhage (IVH) | Yes  No | 24 (7.4)  299 (92.6) | 1 (7.7)  12 (92.3) | 25 (7.4)  311 (92.6) | 0 (0) |
| Asphyxia | Yes  No | 7 (2.2)  316 (97.8) | 1 (7.7)  12 (92.3) | 8 (2.4)  328 (97.6) | 1 (0.3) |
| Intubation | Yes  No | 10 (3.1)  313 (96.9) | 6 (46.2)  7 (53.8) | 16 (4.8)  320 (95.2) | 0 (0) |
| Ventilation | Yes  No | 18 (5.6)  305 (94.4) | 10 (76.9)  3 (23.1) | 28 (8.3)  308 (91.7) | 0 (0) |

**Table S3**. Confusion matrix

By considering class 1 or P for alive neonates and class 0 or N for dead neonates, the confusion matrix was created as Table S3.:

Table S3. Confusion matrix

|  | | **System prediction** | |
| --- | --- | --- | --- |
| **Actual status** | alive | alive | dead |
|  |  | TP | FN |
|  | dead | FP | TN |

In the confusion matrix shown in the table above:

- TP (True Positive) represents that the actual class is positive (alive), and the predicted class of the CBR is also positive (alive).
- FN (False Negatives) represents that the predicted class is negative (dead), but the actual class is positive (alive).
- FP (False positives) represents that the predicted class is positive (alive), but the actual class is negative (dead).
- TN (True Negatives) represents that the actual class is negative (dead), and the predicted class of the model is also negative (dead).

We applied following equations to calculate system performance.

$accuracy= \frac{TP+TN}{TP+TN+FP+FN}$ equation 1

$precision= \frac{TP}{TP+FP}$ equation 2

$sensitivity= \frac{TP}{TP+FN}$ equation 3

$specificity= \frac{TN}{TN+FP}$ equation 4

$F-score= \frac{2*precision*recall}{precision+recall}$ equation 5

$MCC= \frac{TP.TN-FP.FN}{\sqrt{(TP+FP).(TP+FN).(TN+FP).(TN+FN)}}$ equation 6

**Table S4**. confusion matrix on retrospective evaluation with unbalanced data

|  | | **System prediction** | | |
| --- | --- | --- | --- | --- |
| **Actual status** | alive | alive | dead | Total |
|  |  | 319 | 4 | 323 |
|  | dead | 6 | 7 | 13 |
|  | Total | 325 | 11 | 336 |

**Table S5**. confusion matrix on retrospective evaluation with balanced data

|  | | **System prediction** | | |
| --- | --- | --- | --- | --- |
| **Actual status** | alive | alive | dead | Total |
|  |  | 320 | 3 | 323 |
|  | dead | 6 | 7 | 13 |
|  | Total | 326 | 13 | 336 |

**Table S6**. confusion matrix on external validation with unbalanced data

|  | | **System prediction** | | |
| --- | --- | --- | --- | --- |
| **Actual status** | alive | alive | dead | Total |
|  |  | 74 | 0 | 74 |
|  | dead | 2 | 16 | 18 |
|  | Total | 76 | 16 | 92 |

**Table S7**. confusion matrix on external validation with balanced data

|  | | **System prediction** | | |
| --- | --- | --- | --- | --- |
| **Actual status** | alive | alive | dead | Total |
|  |  | 74 | 0 | 74 |
|  | dead | 1 | 17 | 18 |
|  | Total | 75 | 17 | 92 |

**Table S8**. Distribution of qualitative and quantitative features in the prospective dataset for CBR evaluation

| Variables | Values | Survived  (Mean ± SD) | Dead  (Mean ± SD) | All neonates  (Mean ± SD) |
| --- | --- | --- | --- | --- |
| **Quantitative features** | | | | |
| Birth Weight (BW) | 570-4395 | 2529.2±863.2 | 1195.3±881.9 | 2268.3±882 |
| Gestational Age (GA) | 168-287 | 250±27.3 | 206.8±27.3 | 241.6±25.3 |
| **Qualitative features** | | | | |
| **Variables** | **Values** | **Survived**  **N (%)** | **Dead**  **N (%)** | **All neonates**  **N (%)** |
| Preterm birth | Yes  No | 34 (46)  40 (54) | 16 (88.9)  2 (11.1) | 50 (54.3)  42 (45.7) |
| Small for Gestational Age (SGA) | Yes  No | 3 (4.1)  71 (95.9) | 2 (11.1)  16 (88.9) | 5 (5.4)  87 (94.6) |
| Prenatal care | Yes  No | 74 (100)  0 (0) | 18 (100)  0 (0) | 92 (100)  0 (0) |
| Mother disease | Yes  No | 27 (36.5)  47 (63.5) | 7 (38.9)  11 (61.1) | 34 (36.7)  58 (63.3) |
| Multiple birth | Yes  No | 22 (29.8)  52 (70.2) | 7 (38.9)  11 (61.1) | 29 (31.5)  63 (68.5) |
| Respiratory Distress Syndrome (RDS) | Yes  No | 41 (55.4)  33 (44.6) | 14 (77.8)  4 (22.2) | 55 (59.8)  37 (40.2) |
| Steroid therapy | Yes  No | 11 (14.9)  63 (85.1) | 16 (88.9)  2 (11.1) | 27 (29.3)  65 (70.7) |
| Surfactant administration | Yes  No | 3 (4.1)  71 (95.9) | 8 (44.4)  10 (55.6) | 11 (12)  81 (88) |
| Pulmonary hemorrhage | Yes  No | 0 (0)  74 (100) | 4 (22.2)  14 (77.8) | 4 (4.3)  88 (95.7) |
| Congenital malformation | Yes  No | 2 (2.7)  72 (97.3) | 3 (16.7)  15 (83.3) | 5 (5.4)  87 (94.6) |
| Necrotizing EnteroColitis (NEC) | Yes  No | 1 (1.4)  73 (98.6) | 0 (0)  18 (100) | 1 (1.1)  91 (98.9) |
| NEC therapy | Yes  No | 0 (0)  74 (100) | 0 (0)  18 (100) | 0 (0)  92 (100) |
| Sepsis | Yes  No | 56 (75.7)  18 (24.3) | 12 (66.7)  6 (33.3) | 68 (73.9)  24 (26.1) |
| Intra Ventricular Hemorrhage (IVH) | Yes  No | 35 (47.3)  39 (52.7) | 9 (50)  9 (50) | 44 (47.8)  48 (52.2) |
| Asphyxia | Yes  No | 4 (5.4)  70 (94.6) | 1 (5.6)  17 (94.4) | 5 (5.4)  87 (94.6) |
| Intubation | Yes  No | 48 (64.9)  26 (35.1) | 17 (94.4)  1 (5.6) | 65 (70.7)  27 (29.3) |
| Ventilation | Yes  No | 14 (18.9)  60 (81.1) | 16 (88.9)  2 (11.1) | 30 (32.6)  62 (67.4) |

**Table S9.** Scenario of performing tasks in the system

| **Scenario** | **Main task** | **Sub-tasks** |
| --- | --- | --- |
| Create a new account based on your personal information. | Create a new account | 1- Open the main page of the program  2- Select the “registration” option  3- Enter the information required to complete the registration  4- Save the process |
| Select the prediction system, enter a neonate with BW = 2840, GA = 239, preterm delivery, prenatal care, with CHD, IVH, intubation and ventilation, and select 5 results to be displayed by the system | Identify similar cases to the new case | 1. Select prediction system  2- Enter the specifications of the new neonate  3- Select the search button  4- Determining the number of results desired by the user  5- Select the confirmation button |
| Save the entered data as a new case in the case base. | Add a new case to the case base | 1. Select the “add” option  2- Completing the information about the record number, place of birth, LOS predicting and discharge status  3- Select the confirmation button |
| Select the LOS prediction system and enter a neonate with these characteristics: BW = 3220, GA = 248, received prenatal care, with RDS, CHD and sepsis and received ventilation and set 3 results to be displayed by the system. | Identify similar case to the new case | 1. Select LOS prediction System  2- Enter the specifications of the new neonate  3- Select the search button  4- Determining the number of results desired by the user  5- Select the confirmation button |
| Save the entered information as a new LOS case in the case base. | Add a new LOS case to the case base. | 1. Select the “add” option  2- Completing the information about the record number, place of birth, LOS predicting and discharge status  3- Select the confirmation button |
